# Experimental data for cooling rate-dependent properties of Polyphenylene Sulfide (PPS) and Carbon Fiber Reinforced PPS (CF/PPS)

**DOI:** 10.1016/j.dib.2022.108817

**Published:** 2022-12-09

**Authors:** Sota Oshima, Ryo Higuchi, Masaya Kato, Shu Minakuchi, Tomohiro Yokozeki, Takahira Aoki

**Affiliations:** a Department of Aeronautics and Astronautics, Tokyo Metropolitan University, 6-6 Asahigaoka, Hino-shi, Tokyo 191-0065, Japan; b Department of Aeronautics and Astronautics, The University of Tokyo, 7-3-1 Hongo, Bunkyo-ku, Tokyo 113-8656, Japan

**Keywords:** Thermoplastic resin, Mechanical properties, Mechanical testing, Density, Coefficient of thermal expansion

## Abstract

In this study, the cooling rate-dependent properties of polyphenylene sulfide (PPS) and carbon fiber reinforced PPS (CF/PPS) manufactured at different cooling rates (1, 5, and 10 °C/min) are presented. The cooling rate-dependent densities of neat PPS and CF/PPS were determined based on the Archimedes' principle. The coefficients of thermal expansion (CTEs) were determined using a thermomechanical analyzer. The stress–strain curves of neat PPS manufactured at different cooling rates under tensile, compressive, and shear loading were obtained using a universal tester. In addition, the R curves of CF/PPS and the corresponding load–displacement curves are presented under mode I and mode II loading. The experimental data provide useful information for the development of numerical models that depend on both cooling rates and stress triaxiality. In addition, the data can be directly utilized to evaluate the properties and quality of carbon fiber reinforced thermoplastic components in the aerospace, automobile, energy, and civil engineering industries. Detailed experimental results have been presented in a previous study [1].

**Specifications Table**

**Table:** 

Subject	Engineering, Mechanical Engineering
Specific subject area	Carbon fiber reinforced thermoplastics (CFRTP), Mechanics of composite materials, Cooling rate-dependent properties
Type of data	Graph
How the data were acquired	Electro densimeter (MDS-300, Alfa Mirage Co., Ltd.) Thermomechanical analyzer (TMA8311, Rigaku Corp.) Universal tester (AGX-50kNVD, Shimadzu Corp.) Universal tester (AGS-10kNX, Shimadzu Corp.) Strain amplifier (DPM-711B, Kyowa Electronic Instruments) Data logger (EDX-10B, Kyowa Electronic Instruments)
Data format	Raw Analyzed
Description of data collection	Data were collected from specimens manufactured with different cooling rates. Details are described in this article.
Data source location	Department of Aeronautics and Astronautics, Tokyo Metropolitan University, Hino-shi, Tokyo, Japan Department of Aeronautics and Astronautics, The University of Tokyo, Bunkyo-ku, Tokyo, Japan
Data accessibility	Data are available with this article. Repository name: Mendeley Data Data identification number: 10.17632/twzssgtc69.1 Direct URL to data: https://data.mendeley.com/datasets/twzssgtc69/1
Related research article	S. Oshima, R. Higuchi, M. Kato, S. Minakuchi, T. Yokozeki, T. Aoki, Cooling rate-dependent mechanical properties of polyphenylene sulfide (PPS) and CF/PPS, Compos. Part A Appl. Sci. Manuf. 164 (2023) 107,250. https://doi.org/10.1016/j.compositesa.2022.107250

## Value of the Data

• This experimental data provides cooling rate-dependent properties of neat polyphenylene sulfide (PPS) and carbon fiber reinforced PPS (CF/PPS) composites, which are beneficial for understanding the resulting properties of composite structures prepared using carbon fiber reinforced thermoplastics (CFRTPs).
• In addition to the data reported in the research article [1], the densities, CTEs, and detailed data in the mechanical and fracture tests (stress–strain curves and R curves) are presented.
• These experimental data are valuable for researchers, engineers, and manufacturers who i) investigate the fundamental properties of CFRTPs, ii) simulate deformation and failure behavior as well as the manufacturing process of CFRTPs, and iii) design CFRTP structures.
• The densities and CTEs can be directly used to estimate the properties of the CFRTP components and for numerical simulations.
• Tensile, compressive, and shear stress–strain curves as well as R curves can be used to develop elastic, plastic, and failure models, depending on the cooling rates and stress triaxiality.

## 1 Objective

The relationship between fundamental properties and cooling rates of CFRTPs is still debated owing to the lack of experimental data although these properties are required for the structural design using thermoplastic composites [2], [3], [4]. The main objective of the data article is to provide cooling-rate-dependent properties of PPS and CF/PPS composites. Numerous types of properties determined by experiments such as physical (density), thermal (CTEs), mechanical (modulus, yield stress, and strength), and failure (fracture toughness) properties are presented herein. The detailed properties beyond those presented in the related research articles [1] are provided in this data article.

## 2 Data Description

The data contains

**Fig. 1:**
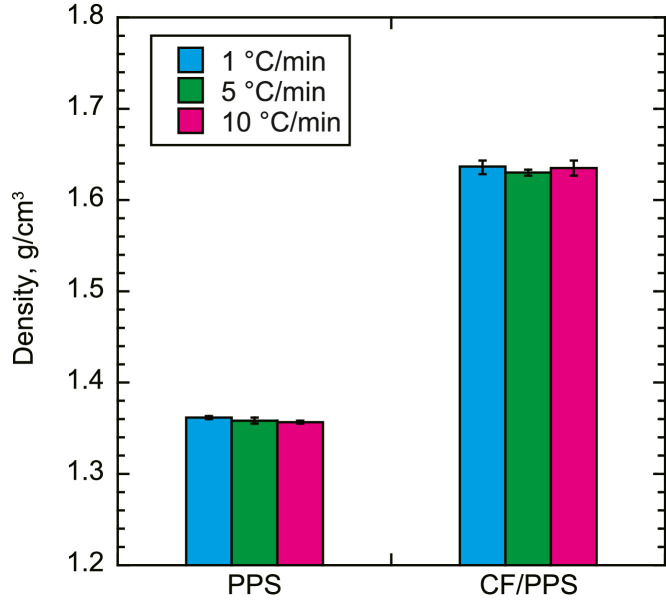
Densities of neat PPS and CF/PPS.
• The CTEs of the neat PPS and CF/PPS in the fiber (0°) and transverse (90°) directions (Fig. 2). The average CTEs between room temperature (23 °C) and glass transition temperature (*T*_g_), *T*_g_ and first melting temperature (*T*_m_^1^), and *T*_m_^1^ and second melting temperature (*T*_m_^2^) are presented in Fig. 2. The characteristic temperatures were determined as described in Ref. [1].

• The densities of the neat PPS and CF/PPS (Fig. 1). The densities were determined based on the Archimedes' principle.

**Fig. 2:**
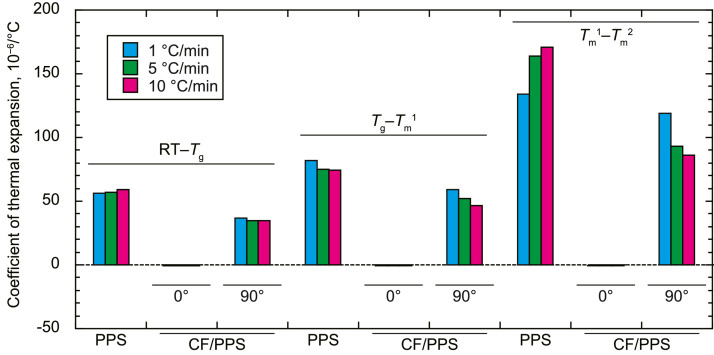
Coefficients of thermal expansion of neat PPS and CF/PPS.
• A representative stress–strain curve of the neat PPS with a cooling rate of 10 °C/min under tensile loading and the corresponding tangential modulus (Fig. 3). The stress–strain curve is given by the nominal stress and nominal strain. The tangential modulus was determined using the moving least squares method.

**Fig. 3:**
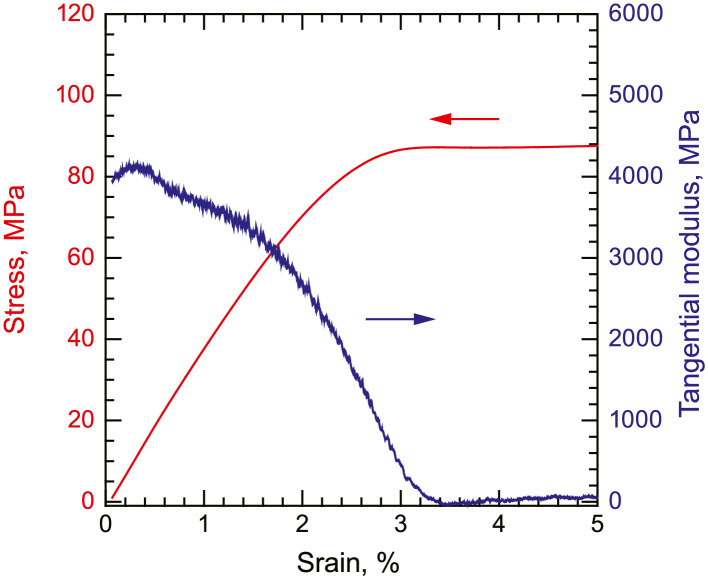
Change in the tangential modulus of neat PPS under tensile loading with a cooling rate of 10 °C/min.
• Representative compressive and shear stress–strain curves of the neat PPS resin (Figs. 4 and 5). Compressive stress–strain curves are given by nominal stress and nominal strain, and shear stress–strain curves are given by shear stress and shear strain.

**Fig. 4:**
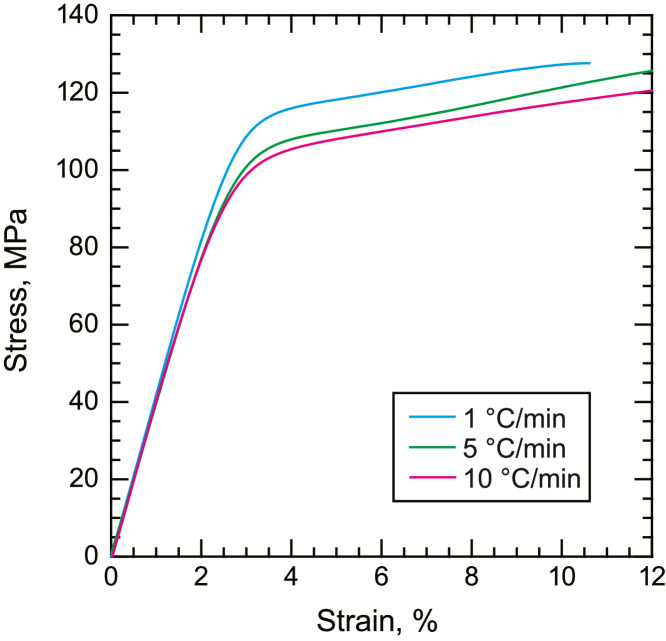
Representative stress–strain curves of neat PPS with different cooling rates under compressive loading.

**Fig. 5:**
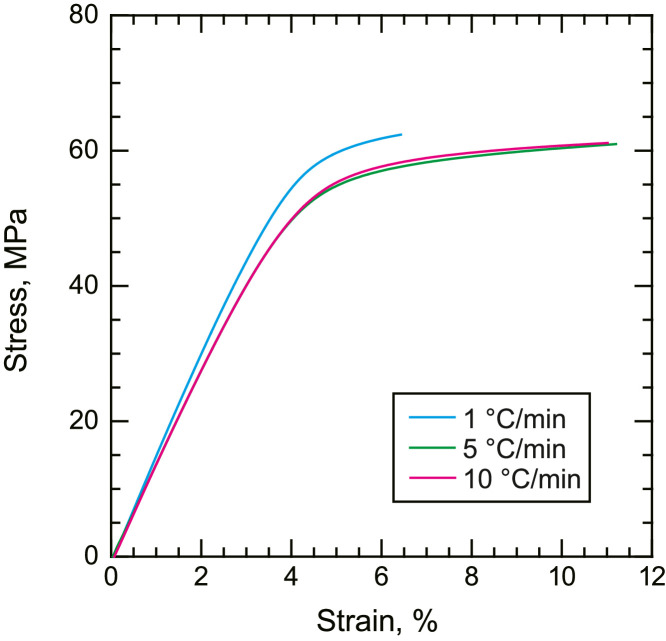
Representative stress–strain curves of neat PPS with different cooling rates under shear loading.
• Representative load–displacement curves of the CF/PPS during mode I (Fig. 6) and mode II (Fig. 7) fracture toughness tests. Representative R curves of CF/PPS under mode I (Fig. 8) and mode II (Fig. 9) loading. Mode I and mode II fracture toughness were obtained using the double cantilever beam (DCB) and end notched flexure (ENF) tests, respectively.

**Fig. 6:**
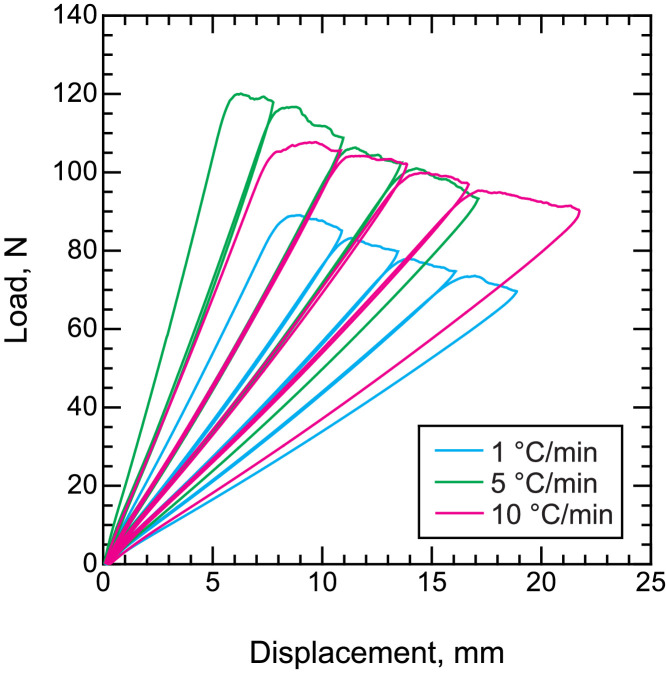
Representative load–displacement curves of fracture toughness tests under mode I loading.

**Fig. 7:**
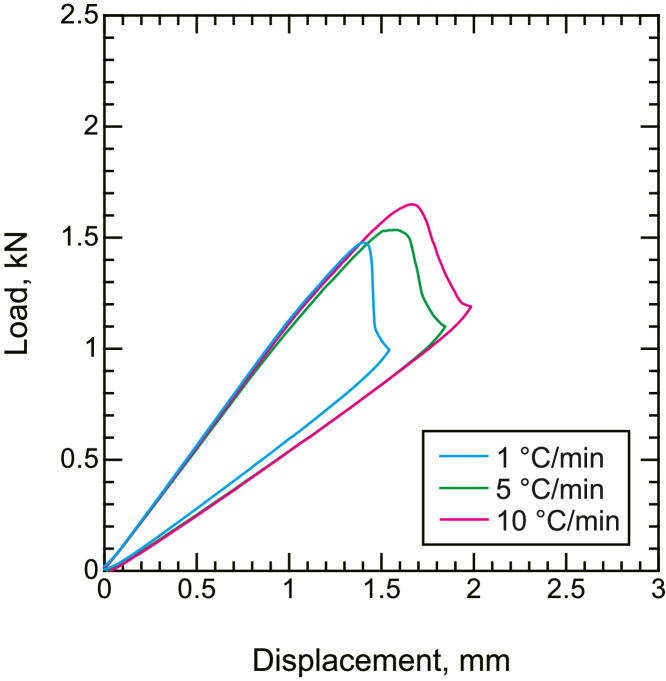
Representative load–displacement curves of fracture toughness tests under mode II loading.

**Fig. 8:**
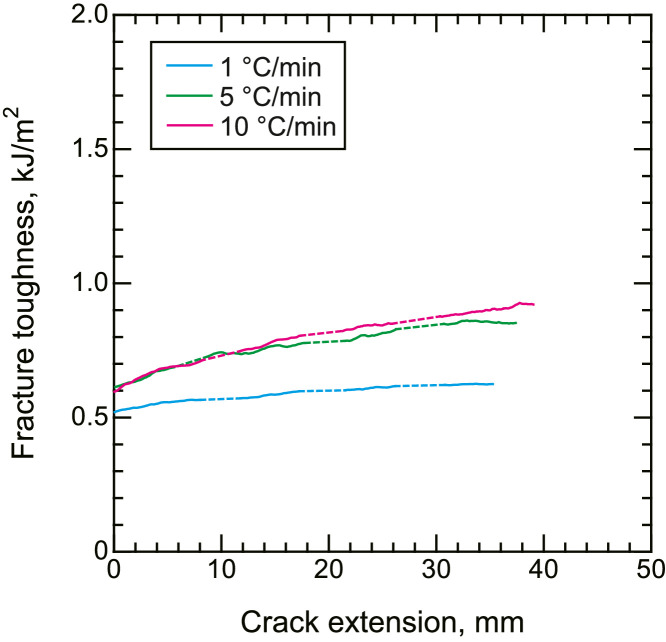
Representative R curves of fracture toughness tests under mode I loading.

All data are presented as a function of the cooling rate (1, 5, and 10 °C/min).

**Fig. 9:**
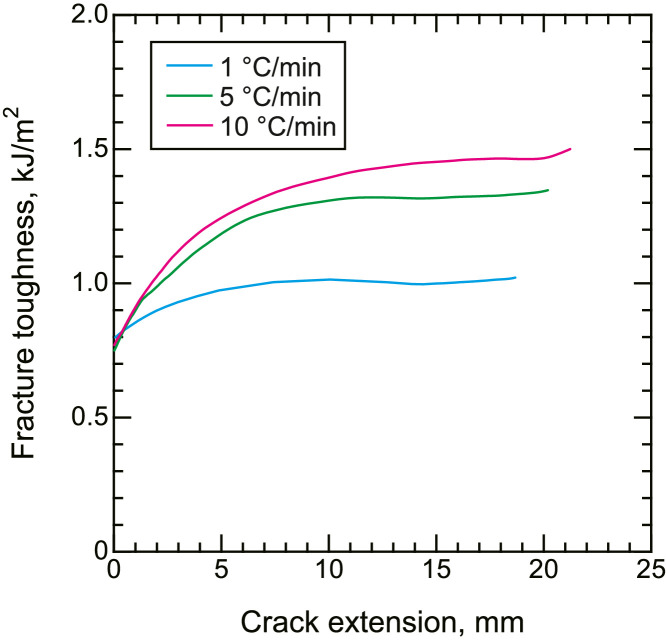
Representative R curves of fracture toughness tests under mode II loading.

## 3 Experimental Design, Materials and Methods

### 3.1 Materials and specimens

The materials used were PPS resin (PPS2000, Toray Industries, Inc.) and CF/PPS (TenCate Cetex® TC1000, Toray Industries, Inc.) consisting of AS4 carbon fibers with a 59% fiber volume fraction. Twenty-four prepreg sheets were unidirectionally stacked to mold the laminates. Neat PPS slabs and CF/PPS laminates were fabricated through hot-press molding. All the specimens were rapidly heated at a heating rate of 10 °C/min from room temperature to 325 °C and held for 20 min. Thereafter, the specimens were cooled at cooling rates of 1, 5, and 10 °C/min with an air-cooling system. During cooling, the pressure was maintained constant at 3 MPa for the neat PPS and 6 MPa for the CF/PPS. The thicknesses of the neat PPS and CF/PPS panels were approximately 3 and 5 mm, respectively. A 50 μm-thick polyimide film, acting as the initial delamination, was inserted in the middle plane of the fracture toughness specimens. Additionally, a pre-crack was introduced with a wedge for the fracture toughness specimens.

The specimens were machined from the neat PPS and CF/PPS panels. Specimens with a length of 10 mm and a width of 5 mm were cut from the panels for CTE measurements. The CTEs in both the fiber (0°) and transverse (90°) directions were measured for CF/PPS. Dog-bone-shaped tensile specimens 170 mm in length and 10 mm in width of the gage section were prepared in accordance with the Type IA specimen in ISO 527–2 [5]. Cubic compression specimens with edge lengths of 12 mm were used for compression tests. V-notch shear specimens were used in the shear tests. The dimensions of the shear specimens were determined in accordance with ASTM D5379 [6]. Strips with a width of 25 mm were used for the mode I and mode II fracture toughness tests. Offcuts of the panels were used to measure the densities.

### 3.2 Measurement of density

The densities of the neat PPS and CF/PPS were measured using an electrodensimeter (MDS-300, Alfa Mirage Co., Ltd.). The densities of the materials were automatically calculated from their weights in air and water. To precisely determine the densities, the data were calibrated by referring to the water temperature. At least three samples were tested under each condition.

### 3.3 Measurement of CTE

The CTEs of the neat PPS and CF/PPS were measured using a thermomechanical analyzer (TMA8311, Rigaku) at a temperature ramp rate of 5 °C/min. A compressive load of 50 mN was applied during measurements. The CTEs of the materials were automatically calculated employing a thermomechanical analyzer using the initial length and elongation of the specimens. One sample was tested under each condition.

### 3.4 Experimental setup of tensile tests

Tensile, compression, and shear tests of the neat PPS were conducted using a universal tester (AGX-50kNVD, Shimadzu Corp.). The crosshead speeds were set to 1, 0.15, and 0.5 mm/min for tensile, compression, and shear tests, respectively. Tensile loading was applied to the dog-bone-shaped tensile specimens using wedge grips. Compressive loading was directly applied at the end of the cubic compression specimens using platens. To reduce friction, the edges of the cubes were lubricated. Shear loading was applied to the V-notch shear specimens using an Iosipescu shear test fixture. Strain gauges were glued to the center of the specimens (KFGS series up to 5%, KFEL series up to 10%, and KFEM series up to 40%, Kyowa Electronic Instruments). The strain signals were amplified using a strain amplifier (DPM-711B, Kyowa Electronic Instruments). The load output from the universal tester and strain output from the strain amplifier were simultaneously collected using a data logger (EDX-10B, Kyowa Electronic Instruments). The stress was calculated by dividing the load by the cross-sectional area of the specimens. All the tests were performed under ambient conditions. At least three samples were tested under each condition, and the representative results are presented for clarity.

### 3.5 Experimental setup of the fracture toughness tests

DCB and ENF tests were performed using a universal tester (AGS-10kNX, Shimadzu Corp.). The crosshead speed was set to 5 and 0.5 mm/min for the DCB and ENF tests, respectively. The load and displacement signals from the universal tester were collected using the attached software. The strain energy release rate of mode I was calculated from the load and displacement using the modified compliance calibration (MCC) method in accordance with JIS K 7086 [7] and ASTM D5528 [8]. Similarly, the MCC method counterpart of mode II loading was also employed for mode II fracture toughness tests, according to Ref. [9]. At least three samples were tested under each condition, and the representative results are presented for clarity.

## Ethics Statement

The authors consciously assure that the manuscript does not involve studies with animals and humans, and follows the ethical requirements for publication in Data in Brief.

## CRediT authorship contribution statement

**Sota Oshima:** Investigation, Writing – original draft, Writing – review & editing, Visualization. **Ryo Higuchi:** Conceptualization, Investigation, Writing – original draft, Writing – review & editing. **Masaya Kato:** Investigation. **Shu Minakuchi:** Supervision, Investigation. **Tomohiro Yokozeki:** Supervision. **Takahira Aoki:** Supervision.

## Declaration of Competing Interest

The authors declare that they have no known competing financial interests or personal relationships that could have appeared to influence the work reported in this paper.

## Data Availability

Dataset for Cooling Rate-Dependent Properties of Polyphenylene Sulfide (PPS) and Carbon Fiber Reinforced PPS (CF/PPS) specimens (Original data) (Mendeley Data)
